# Combined inhibition of heat shock proteins 90 and 70 leads to simultaneous degradation of the oncogenic signaling proteins involved in muscle invasive bladder cancer

**DOI:** 10.18632/oncotarget.5496

**Published:** 2015-11-04

**Authors:** Alice Cavanaugh, Brendon Juengst, Kathleen Sheridan, John F. Danella, Heinric Williams

**Affiliations:** ^1^ Weis Center for Research, Geisinger Health System, Danville, PA, USA; ^2^ Penn State University, Department of Plant Biology, State College, PA, USA; ^3^ Department of Urology, Geisinger Health System, Danville, PA, USA

**Keywords:** heat shock protein 90, heat shock protein 70, muscle invasive bladder cancer

## Abstract

Heat shock protein 90 (HSP90) plays a critical role in the survival of cancer cells including muscle invasive bladder cancer (MIBC). The addiction of tumor cells to HSP90 has promoted the development of numerous HSP90 inhibitors and their use in clinical trials. This study evaluated the role of inhibiting HSP90 using STA9090 (STA) alone or in combination with the HSP70 inhibitor VER155008 (VER) in several human MIBC cell lines. While both STA and VER inhibited MIBC cell growth and migration and promoted apoptosis, combination therapy was more effective. Therefore, the signaling pathways involved in MIBC were systematically interrogated following STA and/or VER treatments. STA and not VER reduced the expression of proteins in the p53/Rb, PI3K and SWI/SWF pathways. Interestingly, STA was not as effective as VER or combination therapy in degrading proteins involved in the histone modification pathway such as KDM6A (demethylase) and EP300 (acetyltransferase) as predicted by The Cancer Genome Atlas (TCGA) data. This data suggests that dual HSP90 and HSP70 inhibition can simultaneously disrupt the key signaling pathways in MIBC.

## INTRODUCTION

Urothelial carcinoma of the bladder (UCB) is the most common malignancy of the urinary tract [1]. It develops along two distinct pathways, each with its unique clinical presentation and molecular pathogenesis [2]. Clinically, UCB presents as either non-muscle invasive disease or muscle invasive bladder cancer (MIBC) [2]. Unlike non muscle invasive disease, MIBC portends a poorer prognosis due to its inherent risk of progressing to metastasis disease resulting in a 1 year median survival [3]. While our understanding of the molecular biology of MIBC has improved significantly, no parallel advancement in treatments beyond cytotoxic chemotherapy has been made [2, 3]. Recent molecular sequencing studies have identified genes and pathways that are key drivers of MIBC [4]. These pathways included p53/Rb (93% altered); histone modifications (89% altered) including acetyltransferases, methyltransferases and demethylases; RTK/Ras/PI(3)K (72% altered), and SWI/SNF (64% altered) [4]. Further network analysis revealed a central role for the heat shock protein 90 AA1 (*HSP90AA1*) gene [4].

Tumor cells utilize multiple oncoproteins to support the tumorigenic process [5] suggesting that anticancer therapeutic approaches should account for each of these strategies in order to improve clinical outcomes. Preclinical trials using HSP90 inhibitors in several cancer models have demonstrated their ability to simultaneously inhibit multiple oncogenic signaling pathways including those involved in the canonical hallmarks of cancer [6]. Tumor cells have been shown to be addicted to HSP90 and high expression has been associated with poor overall survival in non-small cell lung cancer and breast cancer [7, 8]. The ability of HSP90 inhibition to simultaneously inhibit multiple oncogenic signaling pathways had made it an attractive anti-cancer target leading to the development of HSP90 inhibitors, many of which are currently under clinical investigation [9].

HSP90 belong to a class of proteins termed heat shock proteins (HSPs) which are classified according to their molecular weights [10]. Several other HSP families including HSP110, HSP70, HSP60, HSP40 and small HSPs such as HSP27 have been described [10]. In comparison to normal cells, HSPs are expressed at a higher level in cancer cells [11]. Cancer cells usurp the normal function of HSPs to facilitate oncoprotein homeostasis in an increasingly hostile environment [12]. To accomplish this, HSPs ensure the proper folding and assembly of their client protein complexes by a process that is highly coordinated and mediated by number of co-chaperones [13, 14]. Using the steroid hormone receptor as a HSP/client protein model, the process involves the sequential assembly of a HSP40-HSP70-Hop-HSP90 complex [15, 16]. Unfolded client proteins initially bind to a HSP40/HSP70 complex where HSP40 stimulates HSP70-ATP hydrolysis to the HSP70-ADP conformation. This HSP70-ADP conformation favors Hop binding which then recruits HSP90 complex to the growing multi-subunit complex to form the [unfolded client protein-HSP40-HSP70(ADP)-Hop-HSP90] complex. The client protein translocates from the HSP70 complex to the HSP90 complex to complete its maturation [14, 16]. The HSP70 complex dissociates and client protein maturation and activation continues on the HSP90/co-chaperone machinery [17].

HSP90 is an ATP-dependent molecular chaperone that interacts with various co-chaperones (Aha1, p23, HSP organizing protein (Hop), Cdc37) to stabilize it client proteins [6, 18]. This complex is regulated by ATP binding and hydrolysis such that ATP inhibition results in the destabilization of client proteins leading to their ubiquitination and subsequent proteasomal degradation [19]. Over 400 HSP90 client proteins have been identified with an updated list at http://www.picard.ch/downloads/Hsp90interactors.pdf.

Heat shock protein 70 (HSP70) share overlapping cellular functions with HSP90. It is also an ATP-dependent chaperone expressed at low levels in normal or unstressed cells, and inducible under stressful conditions to promote tumor cell survival [20]. Its high expression has also been associated with poor therapeutic outcome in some cancers and conversely, its depletion has led to tumor regression [20, 21]. It forms a complex with its constitutively expressed cognate protein HSC70 [20]. Recent studies showed that dual silencing RNA (siRNA) inhibition of both HSP70 and HSC70 induced proteasome dependent degradation of HSP90 client proteins, G1 cell cycle arrest and tumor specific apoptosis [22]. These observations were not seen when only HSP70 or HSC70 was inhibited by their respective siRNAs [22]. The anti-apoptotic activities of HSP70 are mediated through its interaction with multiple partners in the mitochondrial and lysosomal signaling cascades of apoptosis to protect cells from apoptosis [23]. Other cancer sustaining activities ascribed to HSP70 includes the enhancement of metastasis through interactions with proteins involved in motility and invasion and the promotion of inflammatory conditions in the tumor microenvironment conducive to invasion and angiogenesis [20].

Increased expression of HSP70 is a feature of N-terminal ATPase HSP90 inhibitors and is regarded as a biomarker of drug activity [24]. Some have suggested that this HSP70 overexpression may allow cells to overcome the effects of N-terminal ATPase HSP90 inhibitor drugs when used as monotherapy in clinical trials [25]. We present data showing the effects of HSP90 and HSP70 inhibition alone or in combination on the growth and invasion of human MIBC cells, induction of apoptosis and inhibition of oncogenic signaling pathways in *vitro*. Our studies used the second generation N-terminal ATPase HSP90 inhibitor STA9090 [26] and the HSP70/HSC70 inhibitor VER155008 [27]. We propose that dual targeting of HSP90 and HSP70/HSC70 activity in MIBC cells may be a better therapeutic strategy than HSP90 inhibition alone.

## RESULTS

### Treatment with the HSP90 inhibitor STA9090 induces expression of other heat shock proteins

**Figure 1:**
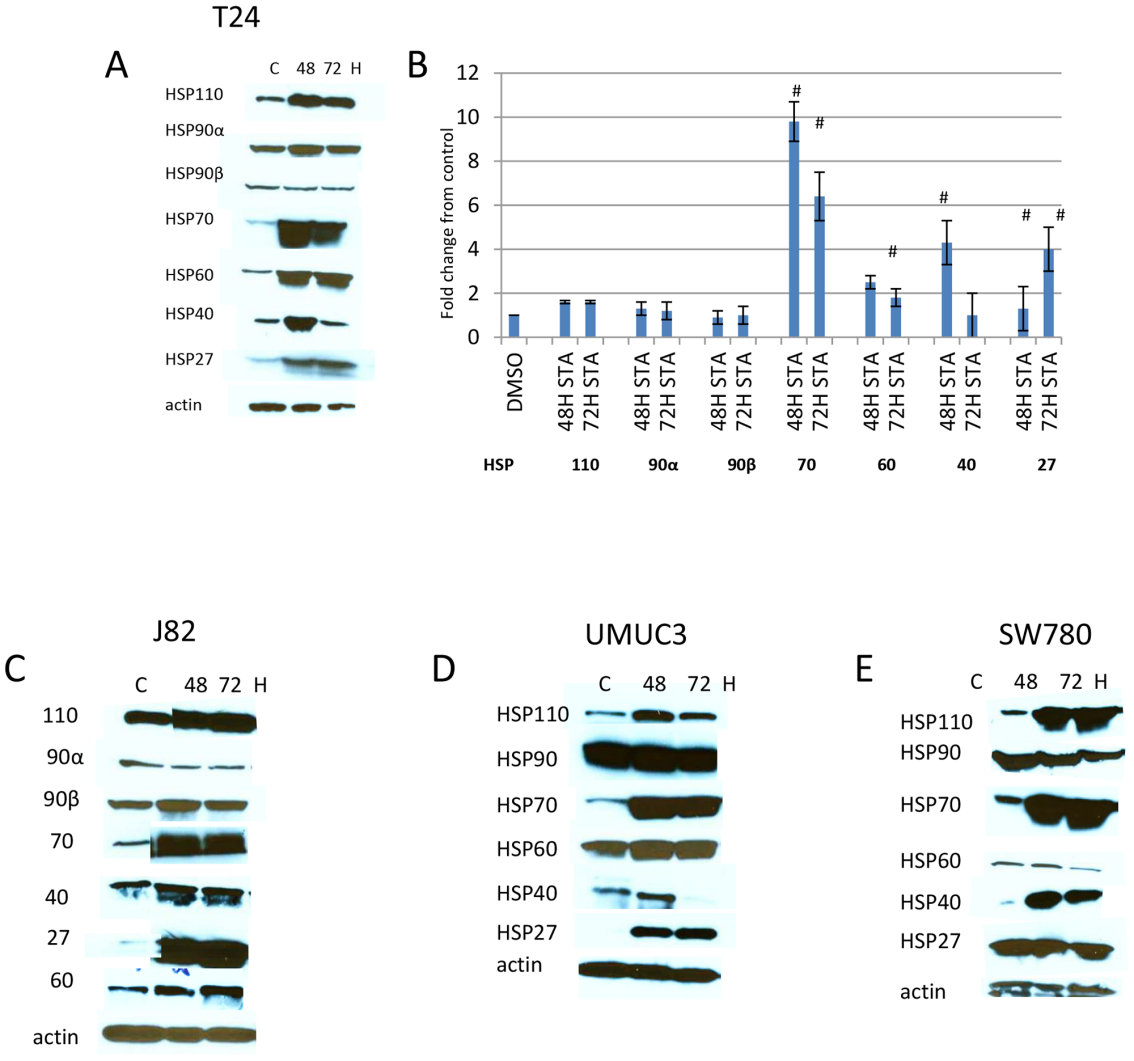
Expression of HSPs in human bladder cancer cells treated with HSP90 inhibitors Bladder cancer cell lines were plated in 100 mm dishes and treated with 1 μM STA9090 for 48 or 72H. Cell lysates were prepared as described in Materials and Methods and fractionated on SDS-PAGE gels. Protein was transferred onto Immobilon P and probed with antibodies to the HSP indicated. **A.** T24 cells, **B.** quantitiation of changes in HSPs expression. Images were quantitated and normalized to actin, results are indicated as fold change over control, # < *p* 0.01, *n* = 3 +/− SD. **C.** J82 cells, **D.** UMUC3 cells, **E.** SW780 cells. Experiments were repeated three times.

The upregulation of HSP70 is a well established feature with N-terminal HSP90 ATPase inhibitors and possibly involved in limiting the efficacy of these drugs [28]. However, to determine whether other HSPs may also be upregulated with N-terminal HSP90 ATPase inhibitors, cells were treated with 1 μM STA for up to 72H and lysates were prepared and fractionated on SDS gels. Western blots were analyzed for the expression of HSPs indicated (Fig 1A). MIBC representing different stages of the disease were used in this study. Both T24 and UMUC3 were derived from muscle invasive only disease (T2), whereas J82 and SW780 were developed from tumors that invaded the perivesical fat (T3) and adjacent viscera (T4) respectively [29]. As expected, increased HSP70 expression after STA treatment was seen in all cell lines (Fig 1A, 1C–1E). However, increased expression of the other HSPs following STA treatment occurred in a cell dependent manner. Expression of HSP60 was elevated in T24, UMUC3 and J82 cells but not in SW780 cells. HSP40 was transiently elevated in T24 cells and UMUC3 cells (compare 48H with 72H), while in J82 and SW780 its expression was maintained for the duration of the experiment. HSP27 expression was induced in T24, UMUC3 and J82cells but not in SW780 cells. The expressions of HSP110, HSP90α and HSP90β were not significantly altered in any of the cell lines.

### Combination therapy with HSP70 and 90 inhibitors was more effective at inhibiting cell growth than monotherapy

**Figure 2:**
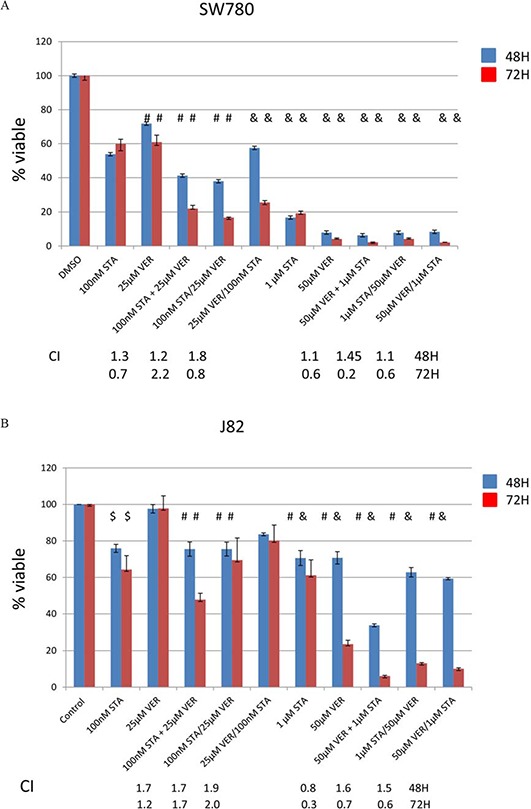

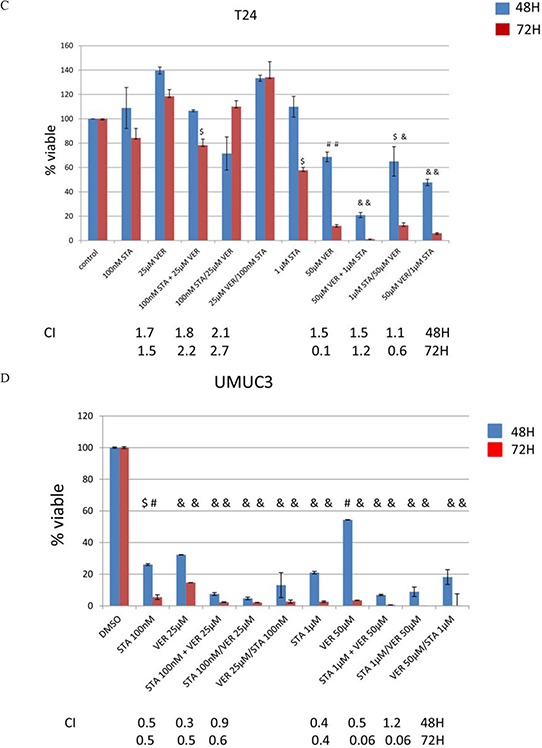
Treatment with HSP90 and/or HSP70 inhibitors are cytotoxic to bladder cancer cells MTT assays of human bladder cancer cells treated with STA9090 and/or VER155008. Bladder cells (20,000/well) were plated into 96 well plates and treated with STA9090 or VER155008 at the concentrations indicated. Where indicated STA9090 and VER155008 were added concurrently (+) or sequentially (/). Forty-eight or 72H from the time treatment was begun cell viability was determined by MTT assays. *N* = 3 +/− SD. $ = *p* < 0.05, #=*p* < 0.01, & = *p* < 0.001. **A-D.** represents the cell lines tested. The combination index listed underneath the dual drug therapy was determined by Chou and Talalay's equation [55].

Since HSP70 was reliably upregulated in all cell lines treated with STA and thought to possess overlapping cellular activities with HSP90, the effect of HSP70 inhibition using VER alone or in combination with STA on MIBC growth was tested. With the understanding that newly synthesized client proteins are initially recognized by HSP40/HSP70 complex and then transferred onto the HSP90 complex to achieve its final folded conformation [14, 16], combination therapy was tested either sequentially or concurrently in order to determine whether the effect on cell growth depended on the order of the drug administration. For the sequential treatment, cells were treated 24 h apart. (We did not test for the optimal treatment interval nor did we have any data to suggest a better treatment interval). Two different drug concentrations were evaluated. The lower concentration consisted of 100 nM of STA and/or 25 μM of VER as monotherapy and/or dual therapy, whereas the higher concentration consisted of 1 μM STA and 50 μM VER. These drug concentrations were based on the IC50 of the drugs for each of the cell lines (Table 1). Cell viability was determined by MTT assays at the times noted following initiation of treatment (Fig 2A–2D). All cell lines showed a decreased in cell viability when treated with each of the HSP inhibitors in a time and dose dependent manner. For the T24 and J82 cell lines, higher doses of both STA and VER were required to achieve a comparable decrease in cell viability seen with the other cell lines (Fig 1B, 1C and Table 1). SW780 displayed intermediate sensitivity to STA and VER monotherapy at the concentrations tested, (Fig 2A and Table 1) while UMUC3 cells were the most sensitive to cell growth inhibition (panel D). Concurrent STA (1 μM) and VER (50 μM) treatment for 72H showed the strongest synergistic effect on cell viability in the SW780, J82 and T24 cell lines as indicated by the CI value of less than one (Fig 2). On the other hand, dual therapy was synergistic at all doses and periods of drug treatment in UMUC cells. Dual therapy also resulted in a left shift of the IC50 values for each drug (Table 1). Significant differences in treatment effects on cell proliferation were determined using the Student's *t* test with a *p* value of < 0.05 deemed statiscally significant. These values are shown in Table 2.

**Table 1:** IC50 value for monotherapy vs dual therapy The concentration of drug where 50% cell death was obtained was compared for mono and dual drug therapy

	Monotherapy		Dual therapy	
Cell line	1C50 STA μM	1C50 VER μM	1C50STA μM	VER μM
T24	0.6	46	0.5	32
SW780	0.4	28	0.1	14
J82	0.8	44	0.1	16
UMUC3	0.1	16	0.05	14

Cells were treated with 1 μM STA9090 and 50 μM VER155008 as mono or concurrent dual therapy and the cell viability determined by MTT assays. The value was determined after 2 hours of treatment.

**Table 2:** Significance of mono therapy vs dual therapy for cell viability *p* values were determined by the Student's *t* test for the combination therapy compared to STA9090 or VER155008 monotherapy

STA monotherapy vs dual therapy				VER monotherapy vs dual therapy		
		*p* value			*p* value	
J82		48H	72H		48H	72H
	100 nM STA + 25 μM VER	NS	0.01	100 nM STA + 25 μM VER	0.001	0.001
	100 nM STA/25 μM VER	NS	0.01	100 nM STA/25 μM VER	0.05	0.001
	25 μM VER/100 nM STA	NS	NS	25 μM VER/100 nM STA	NS	0.001
	1 μM STA + 50 μM VER	0.001	0.001	1 μM STA + 50 μM VER	0.001	0.001
	1 μM STA/50 μM VER	0.01	0.001	1 μM STA/50 μM VER	0.01	0.01
	50 μM VER/1 μM STA	0.001	0.001	50 μM VER/1 μM STA	0.01	0.001
SW780		48H	72H		48H	72H
	100 nM STA + 25 μM VER	0.001	0.001	100 nM STA + 25 μM VER	0.001	0.001
	100 nM STA/25 μM VER	0.001	0.001	100 nM STA/25 μM VER	0.001	0.001
	25 μM VER/100 nM STA	0.001	0.001	25 μM VER/100 nM STA	0.001	0.001
	1 μM STA + 50 μM VER	0.001	0.001	1 μM STA + 50 μM VER	NS	NS
	1 μM STA/50 μM VER	0.001	0.001	1 μM STA/50 μM VER	NS	0.01
	50 μM VER/1 μM STA	0.001	0.001	50 μM VER/1 μM STA	0.01	0.01
T24		48H	72H		48H	72H
	100 nM STA + 25 μM VER	NS	0.05	100 nM STA + 25 μM VER	0.001	0.001
	100 nM STA/25 μM VER	NS	0.05	100 nM STA/25 μM VER	NS	0.001
	25 μM VER/100 nM STA	0.05	NS	25 μM VER/100 nM STA	NS	0.05
	1 μM STA + 50 μM VER	0.001	0.001	1 μM STA + 50 μM VER	0.001	0.01
	1 μM STA/50 μM VER	0.001	NS	1 μM STA/50 μM VER	NS	0.05
	50 μM VER/1 μM STA	0.001	0.01	50 μM VER/1 μM STA	0.01	0.05
UMUC3		48H	72H		48H	72H
	100 nM STA + 25 μM VER	0.001	0.01	100 nM STA + 25 μM VER	0.001	0.001
	100 nM STA/25 μM VER	0.01	0.01	100 nM STA/25 μM VER	0.001	0.001
	25 μM VER/100 nM STA	0.01	0.01	25 μM VER/100 nM STA	0.001	0.001
	1 μM STA + 50 μM VER	NS	0.05	1 μM STA + 50 μM VER	0.001	0.01
	1 μM STA/50 μM VER	NS	0.05	1 μM STA/50 μM VER	0.001	0.001
	50 μM VER/1 μM STA	NS	0.05	50 μM VER/1 μM STA	0.01	0.01

These values were determined from the data shown in Figure 2.

### Effect of STA9090 and VER155008 on cell cycle

**Figure 3:**
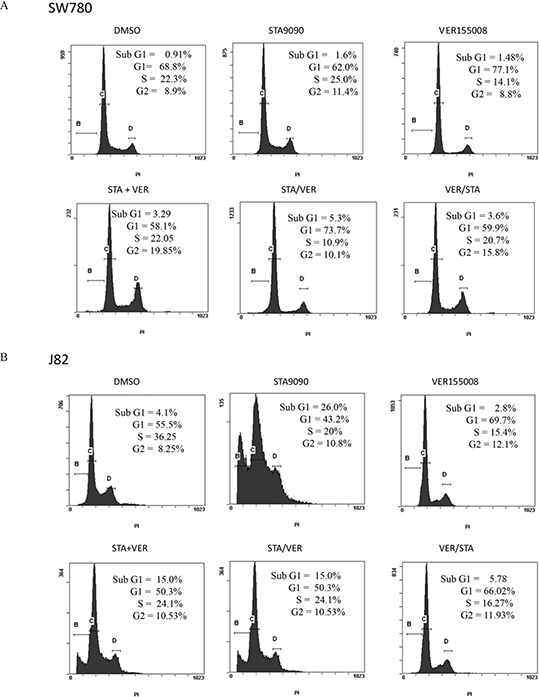

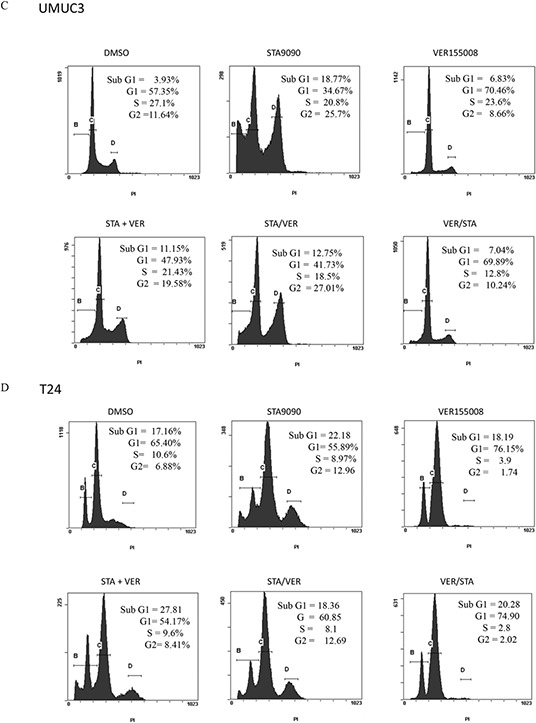

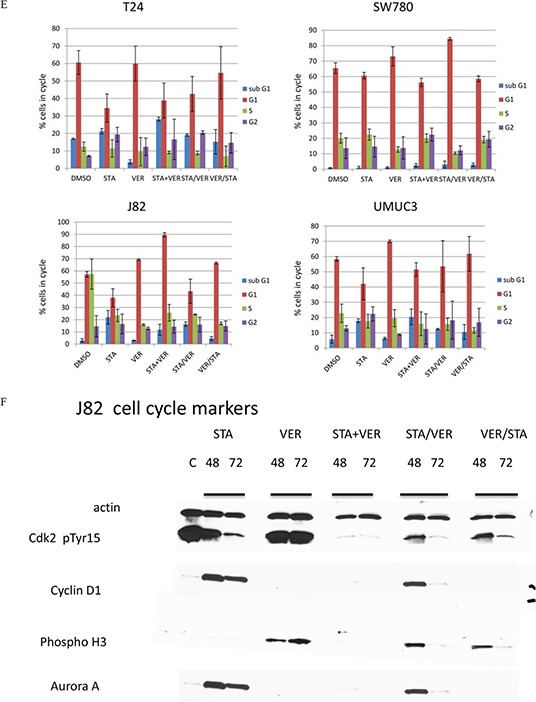
Flow cytometry and cell cycle analysis of bladder cells treated with HSP90 and/or HSP70 inhibitors Bladder cancer cells were treated with 1 μM STA9090 and /or 50 μM VER155008. Drug were added either concurrently or sequentially as indicated. Cells were harvested 48H after the beginning of treatment. The distribution of cells in cell cycle was determined by CXP software, v.2.2. **A.** SW780 cells, **B.** J82 cells. **C.** UMUC3 cells, **D.** T24 cells. These experiments were repeated three times and are quantitated in **E.** mean +/− SD. **F.** J82 cells were treated with 1 μM STA9090 and 50 μM VER155008 as mono or dual therapy for the time indicated. Lysates were prepared as indicated in Figure 1 and Western blots probed for cell cycle markers.

To help elucidate potential mechanisms of these drugs on MIBC lines, the effect of STA and/or VER on cell cycle progression was interrogated by flow cytometry and corroborated with cell cycle phase specific markers by Western analysis. Flow cytometry results of propidium iodide stained cells are shown in Fig 3A–3D. The distribution of cells following STA and/or VER treatments throughout the cell cycle were similar for T24, UMUC3 and J82 cells. STA treatment was associated with an accumulation of cells in sub G1 and G2 while VER treatment resulted in G1 arrest. Concurrent dual therapy showed an accumulation of cells in G2, similar to the STA monotherapy. Sequential combination therapy showed a cell cycle distribution that mirrored the monotherapy results of the first drug in the sequence. All treatments caused a decrease in the number of cells in S phase. STA treatments did not alter SW780 cell cycle progression as compared to control. Interestingly, monotherapy VER treatment arrested cells in G1. The only combination therapy to alter cell cycle progression in SW780 was sequential STA/VER dual therapy leading to G1 arrest. Significance at *p* < 0.05 was determined by the Student's *t test*. The percentage of cells in each cell cycle from three experiments is quantitated and shown in Fig 3E.

To verify the findings from the flow cytometry, cell cycle specific markers were tested by Western analysis using J82 cells after 48H and 72H of mono or combination therapy (Fig 3E). STA treatment resulted in decreased phosphoTyr15 CDK2 expression, no cyclin D1 expression and Aurora A expression consistent with G2 cell cycle arrest. On the other hand, VER treatment led to increased expression of pTyr15 CDK2, phosphorylated histone 3 and cyclin D1 suggestive of G1/M cell cycle arrest. The cell cycle specific marker expression profile of J82 cells treated with combination STA+VER reflected G2/M arrest.

### Effect of combined HSP90 and HSP70 inhibition on apoptosis

Both HSP90 and HSP70 are known to inhibit apoptosis. Therefore, the effect of both STA and VER alone or in combination on apoptosis was examined in the SW780 (least sensitive) and UMUC (most sensitive) cell lines. Apoptosis can be induced either through the extrinsic or intrinsic pathways. The extrinsic pathway is triggered by signals at the cell surface, while the intrinsic pathway is activated through mitochondrial signaling [30]. For this study, cleaved caspase 9 expression was used as a marker of the intrinsic pathway and caspase 8 expression as a marker of the extrinsic pathway [30]. Our results indicate that both STA and VER as monotherapy induced apoptosis through the intrinsic pathway as indicated by the detection of cleaved caspase 9 expression in both SW780 and UMUC3 cells (data not shown). Additionally, VER either alone or in combination activated the extrinsic pathway but not the intrinsic pathway as indicated by the expression of cleased caspase 8 (data not shown).

### Combination HSP90 and HSP70 inhibitor therapy was more effective at inhibiting cell invasion than monotherapy

**Figure 4:**
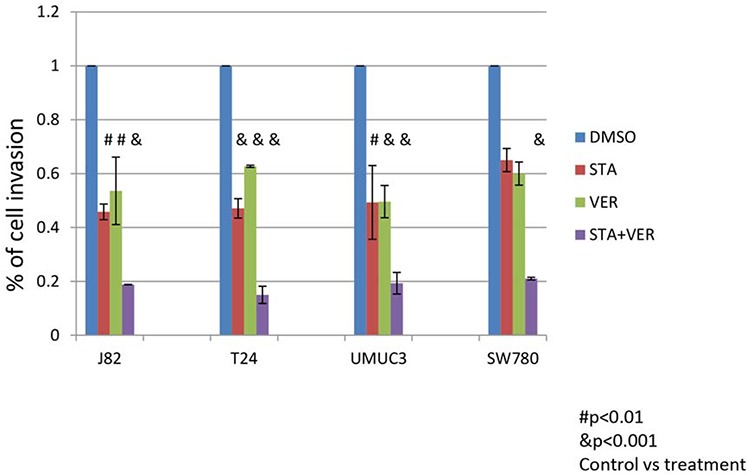
Treatment with HSP 90 and/or HSP70 inhibitors inhibit the ability of cells to invade through Matrigel Bladder cancer cells (2×10^4^) were plated into the upper half of Matrigel Chambers in serum free media. T24, J82 and SW780 cells were treated with 250 nM STA9090 and/or 50 μM VER155008, UMUC3 cells were treated with 100nM STA9090 and /or 50 μM VER155008. The bottom chamber contained EMEM with 10% fetal bovine serum. The cells were allowed to migrate for 22H. The chambers were disassembled and the membranes stained with DiffQuik. Cells that had migrated through the membrane were quantitated. *N* = 3 +/− SD. # = *p* < 0.01, & = *p* < 0.001.

MIBC are naturally invasive cells and this characteristic is associated with poor clinical outcomes. To evaluate the effect of STA and/or VER on the MIBC invasiveness, cells were allowed to migrate through Matrigel for 22 hours. Preliminary experiments indicated that the cell invasion was significantly more sensitive to STA than cell proliferation. A dose response curve using both STA and VER as monotherapy on cell invasion was generated for each cell line (data not shown). Using the cell line specific STA and VER concentrations that would provide 50 to 60% inhibition of cell invasion, the effect of these agents in combination as compared to monotherapy were tested. STA (250 nM) monotherapy resulted in a 40–60% inhibition of cell invasion for T24, J82 cell and SW 780 cells lines, while 100 nM STA showed a 50% inhibition in migration for UMUC3 cells (Fig 4). VER at 50 μM showed approximately 50% inhibition of cell invasion in all the cell lines. MIBC invasiveness was significantly impaired with combination therapy as compared to monotherapy as shown in Fig 4 and Table 3.

**Table 3:** Significance of mono vs dual therapy for cell invasion *P* values determined by the Student's *t* test were determined from the data presented in Figure 5

J82 STA vs dual *p* < 0.01 VER vs dual *p* < 0.05 T24 STA vs dual *p* < 0.01 VER vs dual *p* < 0.01 UMUC3 STA vs dual *p* < 0.05 VER vs dual *p* < 0.05 SW780 STA vs dual *p* < 0.01 VER vs dual *p* < 0.01

### VER155008 treatment does not change HSP 70 expression

Since N-terminal HSP90 ATPase inhibitors induces HSP70 expression [28], we determined the effect of HSP90 and HSP70 inhibition alone or in combination on HSP70 isoforms expression in the cytoplasm or subcellular locations by Western analysis. For combination treatments, cells were treated concurrently or sequentially with STA and VER (24H apart) and harvested at 48H and 72H following treatment. A significant upregulation in the expression of overall HSP70 was seen when cells are treated with STA while VER treatment did not alter total HSP70 expression. Interestingly, total HSP70 expresssion was elevated in cells treated with combined STA and VER treatment regardless of whether they were added concurrently or sequentially (data not shown).

### Effect of heat shock inhibitors on signaling pathways

**Figure 5:**
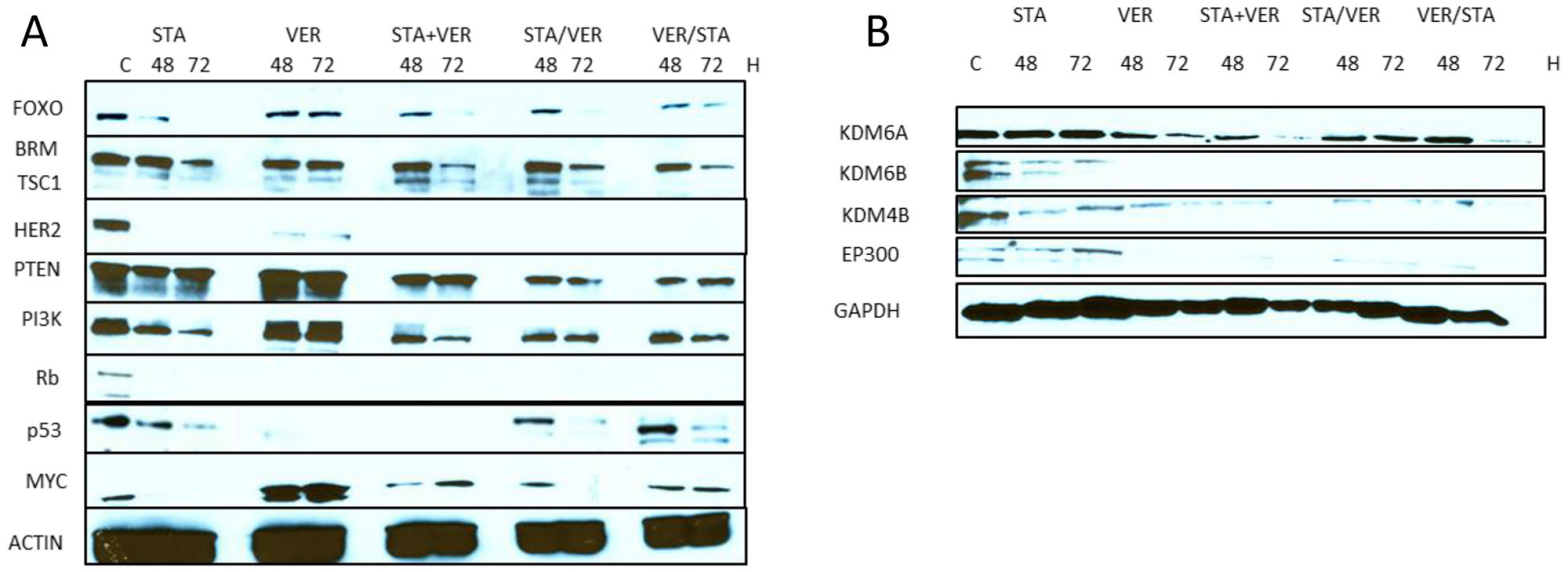
Expression of oncogenic signaling proteins when cells are treated with HSP inhibitors T24 bladder cancer cells were treated with 1 μM STA9090 and /or 50 μM VER155008 as mono or dual therapy as indicated. Lysates were prepared as indicated and Western blots were probed for the proteins indicated. **A.** Proteins that have been reported as frequently mutated in human bladder cancers. **B.** Histone modification proteins: methyltransferases (KDM6A, KDM6B, KDM4B); acetyltransferase (EP300).

A recent report by TCGA Research Network provided a comprehensive molecular characterization of MIBC [4]. This report outlined the genes and pathways altered in MIBC including the p53/Rb, RTK/Ras/PI3K, SWI/SNF and chromatin (or histone) remodeling pathways [4]. We determined the expression of some of the representative pathway genes in T24 bladder cancer cells when treated with HSP90 and 70 inhibitors either as monotherapy or in combination. Results shown in Fig 5A indicated that STA monotherapy and combination therapy effectively inhibited the expression of members of the RTK/Ras/PI3K pathway including pAkt, TSC1, HER2, PTEN and PI3K. Conversely, VER monotherapy had no effect on the expression of any of the proteins tested (data not shown). Results shown in Fig 5A show that expression of members of the chromatin SWI/SNF remodeling pathways (BRM and FOXO) was inhibited more with combination therapy than monotherapy. Interestingly, the expression of both Rb and p53 is totally inhibited with VER monotherapy. Rb expression is also abolished by all combinations of dual therapy. Combination therapy appears to have little effect on expression of p53. The expression of MYC was decreased when cells were treated with STA but increased when cells were treated with VER. Consequentially, MYC expression under conditions of combination therapy was similar to control levels.

The expression of the histone demethylase KDM6A was also determined under conditions of HSP90 and HSP70 inhibition. Unlike many of the signaling proteins described above, KDM6A expression was not affected by STA monotherapy, while VER treatment decreased its expression (Fig 5B). To confirm this, we tested the effect of STA and/or VER on the expression of KDM6B and KDM4B, both histone demethylases reported to be HSP90 client proteins [31]. As expected STA treatment decreased both KDM6B and KDM4B expression, while VER treatment inhibited their expression.

The differential effects of STA and VER treatment was also observed with another member of the histone modification pathway shown to play a role in MIBC, namely EP300, a histone acetyltransferase. STA treatment was not effective in degrading this protein as compared to VER or dual therapy as shown in Fig 5B. Of note, the effect of higher STA concentrations on protein expression was not tested.

## DISCUSSION

An estimated 74,000 new cases of bladder cancer are expected in 2015 along with 16,000 deaths in the United States [1]. At least 30% of these will present with MIBC and if localized at diagnosis, will be managed with radical cystectomy and neoadjuvant chemotherapy to reduce the risk of the development of metastatic disease [3]. Despite our increasing knowledge about the disease and its management, treatment outcomes for advanced bladder cancer have not improved over the last three decades [32]. This may be related to the heterogeneous clinical behavior of UCB in general and MIBC in particular as reflected by the molecular subtypes identified through genome-wide expression and DNA-based analyses [33]. Recently, TCGA described 4 oncogenic signaling pathways perturbed in MIBC and highlighted a central role for HSP90 [4].

HSP90 is a molecular chaperone for several hundred client proteins including protein kinases and transcription factors and plays a role in all cancer hallmarks such that HSP90 inhibition is associated with the simultaneous disruption of multiple tumor survival mechanisms [6], http://www.picard.ch/downloads/Hsp90interactors.pdf. It is therefore not surprising that HSP90 has been an attractive anti-cancer target as compared to other targeted agents focused on inhibiting a single pathway. HSP70 upregulation in serum or peripheral mononuclear blood cells has served as a biomarker of HSP90 inhibitor activity [28]. Unfortunately, the promising anti-tumor activity anticipated from this class of drugs has not been realized in clinical trials [34].

STA is a unique resorcinolic triazolone inhibitor of HSP90 and is currently in clinical trials for a number of human cancers [26]. Preclinical data demonstrated the potency of STA in a range of solid and hematologic tumor cell lines, including those that express mutated kinases that confer resistance to small-molecule tyrosine kinase inhibitors [35]. In comparison to the first generation, N-terminal HSP90 inhibitors such as 17-allylamino-17-demethoxygeldanamycin, STA induced degradation of HSP90 client proteins at nanomolar concentrations, maintained its antitumor activity longer albeit with shorter exposure times and was less toxic. These observations were further validated in solid and hematologic xenograft models, including models of oncogene addiction and no evidence of cardiac or liver toxicity was seen [35].

This study sought to determine the effect of inhibiting both HSP90 and HSP70 activity alone or in combination on MIBC cell lines. Moreover, this study aimed to determine whether adding a HSP70 inhibitor in combination with a HSP90 inhibitor may abrogate the effect of HSP70 upregulation seen with HSP90 inhibition. Finally, this study sought to identify any MIBC oncoproteins proposed by the TCGA data that may be resistant to HSP90 inhibition.

Examination of the HSP40/70/90 cycle reveals a process whereby client proteins are often shuttled to HSP90 for final protein folding [14, 16]. While there is data to suggest that some HSP90 client protein interactions occur without HSP70 involvement, we hypothesized that disrupting the HSP70/90 cycle in MIBC may be a more effective anti-cancer treatment than HSP90 inhibition alone [35]. However, we considered that other HSPs besides HSP70 may also be recruited by cancer cells to compensate for HSP90 inhibition. In this study, along with HSP70 upregulation, there was cell line specific increased expression of both HSP27 and HSP60. While some reports have suggested that HSP27 expression is a poor prognostic factor [36], others have shown HSP60 expression to be favorable [37].

The specific function of HSP70 during HSP90 inhibition in cancer cells has not been clearly elucidated. It is clear that HSP70 can rescue proteins that have been partially denatured, misfolded or become aggregated [38]. This process begins with HSP40 recognizing these aberrant or unfolded proteins and bringing them to HSP70 for proper refolding or folding as appropriate. In this study, HSP40 was elevated transiently during HSP90 inhibition as compared to control and may be helping to coordinate protein rescue with HSP70. However, this function was not formally tested in this study. The binding of HSP40/client protein/HSP70 complex confers the correct conformation for HSP70 ATP hydrolysis as it mediates the protein folding process [39]. HSP70 has been shown to be upregulated in a number of cancers and vital to their survival [40–42]. It supports cancer cell survival by neutralizing the conformational changes in mutant proteins [43] inhibiting cell death pathways [44, 45] and inactivating p53 [46].

Since it appears that protein folding usually proceeds in a coordinated fashion beginning with the HSP70 complex assembly followed by transferring the client protein to the HSP90 cycle [39], we tested whether sequential additional of STA and VER vs concurrent addition influenced cell growth inhibition, promotion of apoptosis and inhibition of MIBC migration. Without any data suggesting the kinetics of the nascent or unfolded protein through these cycles, a lag time of 24 hours between treatments was chosen. However, after noting that concurrent STA and VER treatments as compared to monotherapy STA or VER treatments resulted in synergistic inhibition of both MIBC proliferation and migration while promoting apoptosis, we elected not to pursue the optimal sequencing of the agents in our study.

The effects of STA and VER treatments alone and in combination revealed a cell line specific effect on progression through the cell cycle. STA treatment led to cell cycle arrest in G2 as shown in other studies [47] while VER consistently caused accumulation of cell in G1, in agreement with the results of Powers et al [22]. The combination therapy caused an increase in subG1 and a higher accumulation in G2 when compared to control. With respect to apoptosis, both VER and STA promoted apoptosis through the intrinsic pathway, while VER utilized the extrinsic pathway as well. Treatment with STA cause increased expression of bcl-2, an accepted inhibitor of apoptosis, most likely due to increased expression of HSP70 which has been reported to stabilize bcl-2 [48]. On the other hand, VER monotherapy or combination therapy decreased the expression of both bcl-2 and XIAP (another inhibitor of apoptosis, data not shown).

One of the premises of this study is that increased HSP70 expression may provide a surrogate pathway for cancer cell survival during HSP90 inhibition. However, in this study, we continued to see increased HSP70 expression even in the presence of VER and combined VER and STA treatments. Despite this increased HSP70 expression, cell proliferation, progression through the cell cycle and cell migration were inhibited along with the promotion of apoptosis. This suggested that increased HSP70 expression may be an unreliable marker of treatment efficacy or lack thereof. This may not be surprising as the expression of HSP90 does not decrease with N-terminal HSP90 ATPase inhibitors as well. Therefore, similar to the action of HSP90 inhibitors, it is likely that the biological effect of combination therapy is mediated through impaired function of the ATPase activities of these proteins rather than through their degradation. Interestingly, we observed inhibition of the mortalin and BiP, the mitochondrial and endoplasmic reticulum versions of HSP70, respectively, with HSP90 inhibition. The decrease in expression of these proteins was more enhanced with dual therapy, again suggesting that inhibition of the activity of the cytosolic HSP70 and HSP90 as well as inhibiting the expression of the organelle specific HSP70 isoforms provided a better cell kill and reduced the invasiveness of MIBC. These findings beg the question as to which pool of HSPs is actually driving the cellular processes involved in cell growth, apoptosis and invasion. Since HSP70 has a known role in cellular protein translocation between organelles [38], dissecting the functional roles of these other HSP70 family members in the endoplasmic reticulum and mitochondria may offer more selective anti-cancer therapeutic options going forward.

TCGA identified four aberrant pathways in MIBC [4]. A summary of the effects of STA, VER and combination therapy on members of these pathways is provided in Table 4. STA and not VER monotherapy abolished the expression of proteins of the RTK/Ras/PI3K and SWI/SNF pathways. For the p53/Rb pathway, Rb expression was eliminated by treatment with either drug. The antibody used in this study was capable of recognizing all forms of Rb, i.e. both highly phosphorylated and under phosphorylated. Contrary to other studies where Rb inactivation was associated with hyperphosphorylation of the protein [49] the treatment with either STA or VER totally eliminated Rb expression as did combination therapy. While deletion of Rb has been reported in some bladder cancer cell lines (Sanger Institute Catalogue of Somatic Mutation in Cancer http://www.sanger.ac.uk/cosmic), this mutation has not been reported in T24 cells. Mutation of TP53 is a common mutation in many cancers including urinary bladder cancer [50]. Likewise, it is mutated in T24 cells, AA378C > G (Sanger Institute Catalogue of Somatic Mutation in Cancer http://www.sanger.ac.uk/cosmic). While STA had little effect on expression, VER treatment eliminated any detectable expression. Sequential therapy showed an increase in expression whereas concurrent therapy did not. One may speculate that this is an indirect effect in that sequential therapy may cause degradation of an upstream regulator that may be enhancing turnover of the protein. These results do not address the effects of HSP inhibition on wild type TP53. The effect of HSP inhibition on MYC is also different than that of most of the other oncoproteins reported by the TCGA. In this case, expression of MYC was actually increased by treatment with VER. Again it is possible this is an indirect effect on a upstream regulator of expression.

**Table 4:** Effect of HSP90 /70 inhibition on the expression of proteins reported as altered by the TCGA [4]

Pathway	Inhibitor				
P53/Rb	STA9090	VER155008	STA+VER	STA/VER	VER/STA
p53	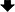	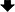	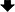	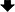	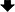
Rb	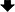	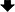	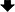	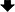	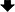
RTK/Ras/PI3K					
HER2	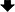	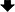	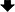	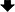	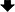
pAkt	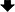	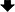	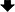	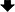	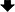
PI3K	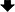	N/C	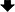	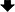	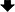
PTEN	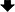	N/C	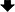	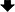	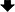
TSC	N/C	N/C	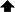 *	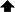 *	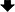
S6K	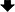	N/C	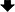	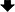	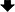
Histone					
Modification					
KDM6A	N/C	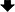	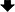	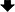	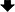
KDM6B	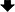	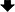	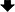	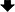	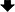
KDM4B	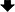	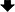	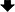	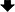	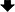
EP300	N/C	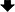	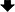	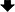	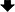
SWI/SNF					
BRM	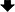	N/C	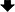	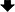	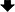

Arrows indicate a decrease or increase in protein expression.

* indicates a transitory elevation after 48H of treatment that is no longer evident after 72H of treatment. N/C indicates no change.

All cell lines tested showed similar results.

One of the interesting findings of this study is the effect of HSP90 inhibition on members of the histone modification pathway. TCGA predicted as central role of the HSP90AA1 gene in this pathway [4]. The stress inducible subunit of HSP90, HSP90α is encoded by this gene [51]. Thus, it was anticipated that along with the aforementioned pathways in MIBC, HSP90 inhibition would be particularly effective. However, STA at the concentrations tested had no effect on KDMA6 expression, a histone demethylase reported to be inactivated in 24% of MIBC cases [4]. In addition, STA treatment had no effect on EP300, a histone acetyltransferase mutated in 15% of MIBC cases. Interestingly, both VER treatment and combination therapy led to their degradation. Wild type KDM6A has been established as a tumor suppressor gene [52, 53]. KDM6A catalyzes the demethylation of tri/di methylated histone H3 at lysine 27 which is associated with transcriptional activation [52] hence this enzyme has global effects to activate either tumor growth or suppression. EP300 in addition has global effects on growth stimulation or suppression due to its ability to acetylate all four core histones [54]. In addition it is known to be a transcriptional activator. While STA treatment has no effect on the expression of this protein, VER treatment caused a significant decrease in EP300 expression. The results indicate a unique role of HSP70 inhibition on the histone modification pathway not predicted by TCGA and stresses the importance of validating the data from next generation sequencing (NGS) studies prior to their clinical application.

Results from this study indicate how data from NGS studies may improve the success of HSP inhibitors in clinical trials. First, it is now possible to confirm or disprove the activity of such therapeutic agents against known or predicted targets in the cancers studied to date. Our study showed that there are a subset of MIBC-associated oncoproteins that are not sensitive to HSP90 inhibition but which are sensitive to HSP70 inhibition, and vice versa, such that the combined inhibition of both HSP70 and HSP90 may lead to a desirable therapeutic outcome. One limitation of this study is that not all of the MIBC associated oncoproteins were expressed in the cell lines tested and thus we were unable to provide a complete compendium of the effects of HSP90 and HSP70 inhibition alone or in combination on all of the reported oncoproteins. Moreover, this study highlights a potential reason for the poor response of HSP90 inhibitor monotherapy in MIBC clinical trials (due its inability to disrupt all the key drivers in MIBC tumorigenesis) and supports the use of combination HSP90 and HSP70 inhibitors therapy. While studies on established cell lines are important, their conclusions need to be verified using *ex-vivo* MIBC. Pre-clinical studies such as these may serve as a template whereby optimal therapies can be identified a *priori* by testing their ability to disrupt all of the signaling pathways suggested by NGS data. Judging from the results of this study, such therapeutic options will likely require multiple agents.

In conclusion, neither HSP90 or HSP70 inhibition alone is an effective treatment option in MIBC. However, dual HSP90 and HSP70 inhibition was superior to monotherapy and appears to be a promising therapeutic alternative capable of simultaneously disrupting the signaling pathways involved in MIBC. The ability of MIBC cells to circumvent the combined inhibition HSP90 and HSP70 inhibition must be anticipated and strategies developed to address this likely consequence in the future.

## MATERIALS AND METHODS

### Growth of bladder cancer cell lines

T24, UMUC3, J82 and SW780 were purchased from ATCC, who verified cell type authenticity. All cell lines were grown in EMEM (Cellgro) supplemented with 10% FBS and penn strep. Cell were harvested by trypsinization, concentrated by centrifugation and lysed in PBS + 5 mM EDTA and 0.5% Triton X100 containing 1 mM Na orthovanadate and Complete protease inhibitor cocktail (Roche).

### MTT assays

Cells were plated at a density of 10,000 cells/well in a final volume of 100 μl in 96 well plates. Twenty four hours after plating cells were treated with STA9090 (MedChem Express), VER155008 (Tocris) or the combination, at the concentrations indicated. In experiments showing sequential addition, the second drug was added 24H after the first addition. Final volume in the 96 well plate was 200 μl. Cells viability was determined by MTT (3- (4,5-dimethylthiazol-2-yl)-2,5-diphenyl tetrazolium bromide assays (Sigma) at the time indicated from the beginning of treatment. Twenty microliters of 5 mg/ml MTT was added to each well and incubated at 37° for 2 H. The media was removed and 100 μl of DMSO added. Plates were read on a SpectraMax 220 plate reader at 570 nm. Significance was determined by the student's *t* test. Significance was set a *p* < 0.05. The combination index (CI) was calculated by the Chou and Talalay protocol [55].

### Western analysis

Protein analysis of cell lysates was determined with the BioRad DC Protein assay. Twenty to thirty micrograms of protein was fractionated on SDS gels and probed with the indicated antibodies: HSP110 1:1000, (Novus Biologicals), HSP90α HRP conjugate (1:20,000) and β, 1:1000, (Enzo Life Sciences); HSP70 1:5000, HSP27 1:1000, p53 at 1:1000 (from StressMarq), HSP60, HSP40 1:1000 (Cell signaling). Other antibodies used in this study, pErk, pAkt, total Erk and Akt, PI3K (110), pTEN, BRM, TSC1, FOXO, HER2, p21 and MYC all from Cell Signaling (1:1000). Antibodies for cell cycle analysis were from Abcam (cell cycle and apoptosis antibody kit.). Loading controls were GAPDH 1:1000 (Santa Cruz) or actin 1:5000 (Sigma). Secondary antibodies were from Jackson Immunolabs and were used at 1:20,000). Detection was by Super Signal WestPico Chemeluminescence (Thermo Scientific). Images were quantitated with Alpha Ease Imaging Unit using Alpha Ease FC Standalone Software.

### Invasion analysis

Invasion analysis was performed in a Corning^®^ BioCoat^®^ Matrigel invasion chamber. Chambers were rehydrated with 500 μl of EMEM (serum free) for 2 HR per the manufacturer's instructions. Cells (2 × 10^4^) were added to the upper chamber in serum free media together with the indicated concentrations of STA9090 or VER155008 alone or in combination. Control wells were treated with DMSO alone. Cells were allowed to migrate toward the chemo attractant in the lower chamber (10% FBS) for 22 hours at 37°. Inserts were removed and stained with DiffQuik. The number of cells that invaded through the Matrigel were counted and normalized to chambers treated with DMSO alone.

### Flow cytometry

Cells were plated in 100 mm dishes and treated with DMSO (control), STA9090, VER155008 and the combination at the concentrations indicated. Treatment was continued for 48H after the last addition. Cells were harvested by trypsinazation and washed with PBS containing 2% FBS. Cells were fixed with 70% ethanol. The fixed cells were treated with RNase A followed by staining with propidium iodide. The distribution of cells in each cycle was determined by flow cytometry in a Cytomics FC500 flow cytometer. Analysis was performed with CXP v 2.2 (Beckman Coulter)
